# Draft Genome Sequence of *Streptomyces* sp. Strain RKCA744, Isolated from the Arauca River, Colombia

**DOI:** 10.1128/mra.00736-22

**Published:** 2022-09-26

**Authors:** Christopher Cartmell, Alyssa L. Grunwald, Carolina Arango, Noelle J. Duncan, Bradley A. Haltli, Luis E. Diaz, Russell G. Kerr

**Affiliations:** a Department of Chemistry, University of Prince Edward Island, Charlottetown, PEI, Canada; b Department of Biomedical Sciences, Atlantic Veterinary College, Charlottetown, PEI, Canada; c Nautilus Biosciences Croda, Charlottetown, PEI, Canada; d Universidad de La Sabana, Chia, Colombia; University of Southern California

## Abstract

*Streptomyces* sp. strain RKCA744 was isolated from sediment collected from the Arauca River, Colombia.

## ANNOUNCEMENT

In continuing bioprospecting efforts to discover new bioactive natural products, sediment (ca. 400 g) was collected from the banks of the Arauca River located at 7.07263889°N, 70.82741667°W, at a water depth of 50 cm. The bacterium was isolated by dilution plating as described previously (1). BLASTN (v. 2.13.0) analysis of the full-length 16S rRNA gene sequence extracted from the genome sequence of *Streptomyces* sp. strain RKCA744 (1,527 bp) indicated that the closest match in the GenBank 16S rRNA gene sequence database was Streptomyces hygroscopicus (100% identity; accession number NR_043379.1) (2, 3). To further interrogate the biosynthetic potential of RKCA744 biosynthetic gene clusters (BGCs) within the genome of this organism, we obtained a draft whole-genome sequence.

*Streptomyces* sp. strain RKCA744 was cultured in Difco ISP2 broth at 30°C with shaking at 200 rpm. DNA was isolated using the DNeasy UltraClean microbial kit (Qiagen) following the manufacturer’s protocol, and 16 μg of high-quality genomic DNA was obtained. Library preparation and Pacific Biosciences RS II DNA sequencing were performed by McGill University and the Génome Québec Innovation Centre using a standard PacBio protocol for sheared large-insert (20-kb) libraries (4) and a PacBio RS II single-molecule real-time (SMRT) platform with P4-C2 chemistry (Pacific Biosciences, USA). Sequencing was performed using one SMRT cell, which generated a total of 221,223 raw subreads (423,176,179 bp) with an average length of 1,913 bp and a GC content of 72.1%. The raw reads were filtered for quality using the SMRT Analysis software (version 2.3.0) (5) with a minimum subread length of 500, a minimum polymerase read quality of 0.75, and a minimum polymerase read length of 100. *De novo* genome assembly of the filtered reads was performed by the sequencing facility using the Hierarchical Genome Assembly Process (HGAP2)/Quiver protocol (5). Assembly resulted in the generation of 392 contigs with a total assembly length of 9,759,387 bp. The longest contig was 164,905 bp, the *N*_50_ was 40,528 bp, and sequencing coverage of the assembled genome was 43×.

The *Streptomyces* sp. RKCA744 draft genome was annotated using the Prokaryotic Genome Annotation Pipeline (6) yielding 7,860 predicted protein-coding sequences and 61 tRNAs (3 rRNA operons) from all contigs.

AntiSMASH (v. 6.1.1) was used to identify natural product BGCs. A total of 62 putative BGCs were identified and included putative siderophore (3), ectoine (2), butyrolactone (1), arylpolyene (1), homoserine lactone (1), terpene (6), nonribosomal peptide (NRP; 10), polyketide (PK; 24), and hybrid NRP/PK (8) BGCs. Several BGCs exhibited a high percentage of similarity to several known gene clusters including isorenieratene (85% similarity) (7), ectoine (100% similarity) (8), ochronotic pigment (75% similarity), hopene (76% similarity) (9), pristinol (100% similarity) (10), desferrioxamine B (100% similarity) (11), and geosmin (100% similarity) (12).

### Data availability.

This whole-genome sequencing project has been deposited at GenBank under the accession number JAMYEP000000000. Raw sequencing data sets, including the longest filtered subreads, filtered subreads, and circular consensus sequences (CCSs), have been registered in the NCBI SRA database under the accession number SRP389029 with the BioProject accession number PRJNA853303.
